# The association between parents phubbing and prosocial behavior among Chinese preschool children: a moderated mediation model

**DOI:** 10.3389/fpsyg.2024.1338055

**Published:** 2024-03-18

**Authors:** Dasheng Shi, Yongqi Xu, Lin Chu

**Affiliations:** ^1^School of Education, Minzu University of China, Beijing, China; ^2^Teachers’ College, Beijing Union University, Beijing, China

**Keywords:** Chinese preschool children, parents phubbing, prosocial behavior, closeness child–parent relationship, authoritative parenting style

## Abstract

**Introduction:**

The popularization and widespread use of smartphones and other electronic devices have led to the occurrence of “parents phubbing”, which may have a negative impact on child-parent relationship and preschoolers’ prosocial behavior.

**Methods:**

To clarify this process, a questionnaire survey was conducted with 3,834 parents from 20 kindergartens in Zhuhai, China. This study examined the relationship between between parents phubbing, closeness child-parent relationship, authoritative parenting style and children’s prosocial behavior.

**Results:**

According to the study, we found a significant negative correlation between parents phubbing and preschoolers’ prosocial behavior. Closeness child-parent relationship mediated between parents phubbing and preschoolers’ prosocial behavior through mediation effects analysis. In other words, parent phubbing was negatively associated with closeness child-parent relationship, which in turn predicted less child prosocial behavior. In addition, authoritative parenting styles have a moderating effect. As the level of authoritative parenting style increases, the negative impact of parent phubbing on the prosocial behavior of preschool children is attenuated.

**Discussion:**

This study contributes to the understanding of the relationship between parents phubbing and prosocial behaviors of preschool children, as well as the internal mechanisms at work. Practically, the study suggests that parents should reduce the incidence of phubbing in their contact with their children and, at the same time, work to improve the child-parent relationship and promote the development of prosocial behaviors in children.

## Introduction

Prosocial behavior refers to an individual consciously engaging in behaviors that benefit others in a social interaction situation, encompassing sharing, helping, cooperating, and comforting (Carlo, 2014). Children generally demonstrate prosocial behaviors of helping others with simple tasks after the age of one (Warneken and Tomasello, 2007). During early childhood, children’s prosocial behaviors will gradually increase, such as sharing objects with others and comforting others (Dunfield and Kuhlmeier, 2013). Prosocial behavior in childhood is an outward manifestation of the development of personality traits and moral character, and is an important reflection of social development (Wang and Wang, 2021). Children who frequently exhibit pr-social behavior can gain higher peer acceptance, have good interpersonal relationships, and thus reduce problematic behaviors such as social withdrawal and aggression (Liu et al., 2022). In addition, prosocial behavior has a special role in human development and is morally important for the formation of a code of conduct in the process of integrating into complex social environments (Wu and Li, 2015).

The preschool period is the basic stage of children’s social development, and it is also the period of their most rapid development. The development of prosocial behavior in children is taken seriously in many countries around the world. For example, the Head Start program in the United States, which is one of the major projects of the early education program of the United States Government, emphasizes the social–emotional aspects of children’s development (McCrae et al., 2016). The Early Years Foundation Stage in the United Kingdom also states the objectives and guiding principles for promoting children’s social development (Forrester et al., 2022). Family Support Services in Germany aims to promote the social–emotional development of children by providing various services such as family education programs and parent–child activities (Ris et al., 2020). In China, preschool children’s prosocial behavior has received extensive attention from the government and society. China’s various preschool education regulations and related documents refer to the specific content and educational goals of children’s prosocial behavioral development. For example, the Outline of Guidance for Kindergarten Education (for Trial Implementation), issued in 2001, clearly states that the educational objectives of the social field for young children include: “to be willing to interact with others, to learn mutual assistance, cooperation and sharing, and to be compassionate; and to understand and abide by the basic rules of social behavior in daily life” (Developed by the Ministry of Education of the People’s Republic of China, 2001). The Learning and Development Guidelines for Children aged 3 to 6, issued in 2012, mention that the learning and development goals in the social domain for young children include “being willing to interact with others, being able to get along with peers, enjoying and adapting to group life, and abiding by basic norms of behavior” (Li and Feng, 2012). *The Outline for the Development of the Chinese Child* (2021–2030), issued in 2021, emphasizes the need to create friendly, equal and respectful teacher-student and classmate relationships, as well as to enhance parent–child interactions and establish equal and harmonious child–parent relationships (National Bureau of Statistics, 2021). It can be seen that the development of prosocial behavior in preschool children has received extensive attention from the Chinese government and society.

### Parents phubbing and children’s prosocial behavior

In today’s era of rapid development of new media technology, all kinds of new media are emerging, and the number of families with smart phones, voice assistants and tablet computers is increasing (Chaibal and Chaiyakul, 2022). Information technology has brought about a change in people’s lifestyles and also a change in interpersonal communication, with the popularization and widespread use of smartphones and other products leading to a kind of “phubbing” that is not conducive to interpersonal communication and interaction. The word phubbing, which first appeared in Australia’s Macquarie Dictionary, is a new type of word synthesized from phone and snubbing, which came about as a way to get people to put down their cell phones and get back to talking to each other again. According to Aagaard’s (2020) research, phubbing refers to a social phenomenon in which an individual’s eyes are glued to a mobile device while interacting with another person, ultimately leading to a breakdown in conversation or communication. Chinese scholar Hong et al. (2019) argued that parents phubbing usually occurs in the home environment, where parents appear to be distracted by cell phone use in the presence of their children. Parents phubbing may affect the development of prosocial behavior in preschool children (Hong et al., 2019).

Specifically, when parents are too immersed in their cell phones, computers, or other screen devices, they neglect to interact with their children, who may feel neglected and isolated. This lack of attention can lower a child’s self-esteem and reduce their motivation for prosocial behavior (Wang et al., 2022). Piaget’s theory of cognitive development suggests that during the concrete operations stage, children begin to be able to take into account the views and feelings of others (Piaget, 2008). When parents are actively involved in their children’s lives and show concern for them, children are more likely to develop positive prosocial behaviors, such as the ability to share, cooperate, and care for others (Hu and Feng, 2022). Additionally, the preschool years are a critical time for children to learn social skills, and if parents are constantly looking down at their phones, they may not be able to provide their children with enough opportunities for social interaction (Niu et al., 2020). Prosocial behavior often involves the ability to express, share, and communicate emotions, and if parents rely too much on electronic devices in front of their children, the children may lack opportunities to learn to express and communicate their emotions. This may result in children having difficulty understanding and responding to the emotions of others, reducing their level of prosocial interaction with peer (Xu and Xie, 2023). Thus, parents phubbing may have a negative impact on preschoolers’ prosocial behavior, i.e., the higher the level of parents phubbing, the worse the development of preschoolers’ prosocial behavior may be.

### The mediating role of closeness child–parent relationship

Bronfenbrenner points out that in ecosystem theory, microsystems are the systems that are most closely and directly linked to the individual, including families, schools, communities, etc. (Bronfenbrenner, 1975). The family in the microsystem is a complex whole in which the child–parent relationship is recognized as a key factor influencing the development of young children (Lussier et al., 2002). Child–parent relationships are usually categorized into close, conflictual and dependent child–parent relationships (Zhu et al., 2022). Closeness child–parent relationship is a model of parenting based on deep emotional connection, open communication, mutual support, respect for individual differences, sharing of time and experiences, and providing a sense of security (Rinaldi et al., 2023). Closeness child–parent relationships may mediate the link between parents phubbing and children’s prosocial behavior. Firstly, parents phubbing may be detrimental to the development of a closeness child–parent relationship. When parents look down at screens, they tend to spend less time interacting with their children, which can lead to less child–parent communication and intimacy (Hefner et al., 2019). Additionally, closeness child–parent relationships require a commitment of time and attention, and screens distract parents from connecting with their children on a deeper level (Sundqvist et al., 2020). Secondly, closeness child–parent relationships further influence children’s prosocial behavior. Closeness child–parent relationships provide children with the emotional support and security they need, and this support helps to develop children’s trust and emotional well-being, making them more willing to develop positive prosocial relationships with others (Saral and Acar, 2021). Furthermore, closeness child–parent relationships emphasize open and honest communication, enabling children to better understand the feelings of others and to respond positively, an ability that underpins the development of prosocial behavior in children (Calatrava et al., 2023). Thus, the closeness child–parent relationship may play some role in the relationship between parents phubbing and preschool children’s prosocial behavior. In other words, parents phubbing influences preschool children’s prosocial behavior by affecting the closeness child–parent relationship.

### The moderating role of authoritative parenting style

The authoritative parenting style is a parenting style that fosters autonomy and social skills through positive child–parent communication and educational guidance (Nie et al., 2022). This type of parenting has a positive impact on the establishment of child–parent relationship and the development of children’s prosocial behavior (Ontai and Thompson, 2008). Thus, we can speculate that authoritative parenting styles perhaps moderated the relationship between parents phubbing, closeness child–parent relationship and prosocial behavior in preschool children. Firstly, the authoritative parenting style emphasizes parental understanding and communication, as well as the need to meet the children’s emotional needs, which contributes to a close child–parent relationship (Mercer, 2011; Lavric and Naterer, 2020). It is clear from the above that parents phubbing is detrimental to the development of closeness child–parent relationship, which in turn further influences the development of children’s prosocial behavior. However, authoritative parenting styles contribute to closeness child–parent relationships. Therefore, authoritative parenting removes to some extent the negative impacts of parents phubbing and promotes the establishment of closeness child–parent relationship and the development of children’s prosocial behavior. In other words, authoritative parenting styles perhaps moderated the relationship between parents phubbing and closeness child–parent relationship, closeness child–parent relationship and preschoolers’ prosocial behavior.

Secondly, authoritative parenting style emphasizes children’s learning to share, cooperate and care for others, and promotes social interaction and emotional expression, which also contributes to the development of prosocial behaviors among preschoolers (Winsler et al., 2005; Zhao et al., 2023). We have pointed out above that parents phubbing has a negative impact on the prosocial behavior of preschool children. However, authoritative parenting style promotes the development of prosocial behavior in preschool children. Thus, authoritative parenting styles may have moderated the relationship between parents phubbing and preschool children’s prosocial behavior.

### The present study

**Figure 1:**
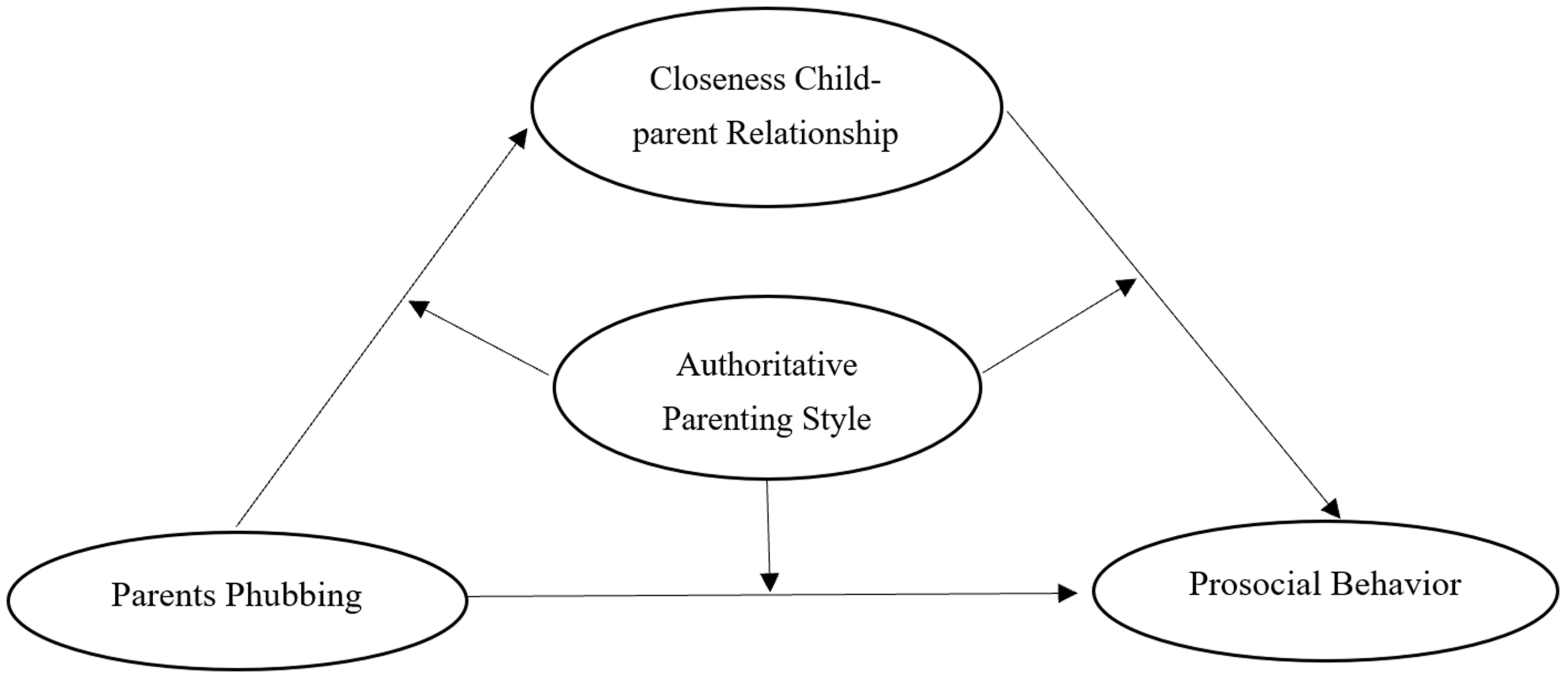
The hypothetical model.

Research on preschoolers’ prosocial behavior has focused on two main areas: first, the developmental characteristics of preschoolers’ prosocial behavior itself. For example, it has been noted that preschoolers’ prosocial behavior increases with age and is directed more toward same-sex peers as they grow older (Song et al., 2021). At the same time, it has also been noted that most of the prosocial behaviors of preschool children are not reinforced in a timely manner (Cigala et al., 2015). Secondly, the influencing factors of prosocial behavior in preschool children. Some studies have pointed out the possible influence of factors such as gender, family economic status, and parents’ education level on the development of prosocial behavior in preschool children (Schachner et al., 2018; Tavassoli et al., 2023). In conclusion, previous studies have mainly focused on the characteristics of preschool children’s own development of prosocial behaviors, as well as studies of related influencing factors (Kokanovic and Opic, 2018; Hu and Feng, 2022; Ozbal and Gonen, 2023). However, fewer studies have focused on the impact of parents phubbing on the prosocial behavior of preschool children. In the current era of rapid development of new media, parents phubbing is a common phenomenon in family life, and this phenomenon has an important impact on the development of preschool children’s prosocial behavior. Therefore, the present study focused on the impact of parents phubbing on preschool children’s prosocial behavior, incorporating variables such as closeness child–parent relationship and authoritative parenting styles, and developed a mediated moderation model (see Figure 1).

In addition, this study proposes the following hypothesis: Parents phubbing has a negative influence on preschoolers’ prosocial behavior (H1); Closeness child–parent relationship mediates between Parents phubbing and Children’s prosocial behavior (H2); Authoritative parenting style moderates the relationships among Parents phubbing, Closeness child–parent relationship, and Children’s prosocial behavior (H3).

## Methods

### Participants

The study conducted an online survey of parents of 3,834 kindergarten preschoolers from 20 kindergartens in Zhuhai, China. To ensure the questionnaire quality, we first contacted the kindergarten directors to explain our research intentions and obtained their support. In addition, we provided instructions to the kindergarten parents and obtained their support. By anonymously filling out the questionnaire, a total of 3,483 valid questionnaires were finally collected according to the rejection criteria such as missing answers, reverse question items, and the same answers to consecutive questions. The sample composition was as follows: among the participants, there were 702 fathers, accounting for 20.16%, and 2,781 mothers, accounting for 79.84%; among the participants’ children, there were 1767 boys, accounting for 50.73%, and 1716 girls, accounting for 49.27%; there were 909 only child, accounting for 26.1%, and 2,574 non-only child, accounting for 73.9%; 80 were single-parent families, accounting for 2.3%, and 3,403 were non-single-parent families, accounting for 97.7%; among the children, 904 were 3 years old, accounting for 25.95%, 1,044 were 4 years old, accounting for 29.97%; 1,207 were 5 years old, accounting for 34.65%, and 328 were 6 years old, accounting for 9.42%. All measurements and procedures were permitted by the Institutional Review Board (IRB) of the first author’s institution (Table 1).

**Table 1:** Composition of participants.

Boys	1767 (50.73%)	3 years	904 (25.95%)
Girls	1716 (49.27%)	4 years	1044 (29.97%)
Only child	909 (26.1%)	5 years	1207 (34.65%)
Non-only child	2574 (73.9%)	6 years	328 (9.42%)
Single-parent families	80 (2.7%)	Total	3483
Non-single-parent families	3403 (97.7%)		

Only child, only one child in the family; Non-only child, more than one child in the family.

### Measures

The questionnaire items in this study were translated and adapted from prior studies. For the translation, we had two doctoral students in the field of educational psychology translate separately and then compare and revise. We then invited two educational psychologists to review them. In addition, to validate the questionnaire in, a small preliminary test was conducted before its formal implementation.

### The parents phubbing

The parents phubbing scale is adapted from Roberts and David’s phubbing scale, which is a one-dimensional scale with nine questions (Roberts and David, 2016). Questions include, “I look at my cell phone while eating with my child” “I often look at my cell phone when talking with my child” “I hold my cell phone in my hand when I am with my child” and so on etc. The questionnaire was rated on a scale of 1–5, from 1 “very inconsistent” to 5 “very consistent,” and the average score was taken as the final score, with the higher the score the more serious the parents phubbing. The Cronbach’s alpha coefficient for this questionnaire was 0.767.

### The closeness child–parent relationship

Closeness child–parent relationship was adopted from Pianta’s Child–Parent Relationship Scale (Pianta and Virginia, 1992), which consists of three dimensions: conflictual child–parent relationship, closeness child–parent relationship, and dependent child–parent relationship. The closeness dimension was selected for this study with a total of 10 questions. The questions include “I have a close relationship with my child” “I can easily empathize with my child” “My child shares his/her things with me” and so on. The questionnaire was rated on a scale of 1–5, from 1 “not at all” to 5 “very much,” and the average score was taken as the final score, with the higher the score the higher the level of closeness child–parent relationship. The Cronbach’s alpha coefficient for this questionnaire was 0.710.

### The preschoolers’ prosocial behavior

Preschoolers’ prosocial behavior was measured using the 5-item prosocial behavior subscale of the Goodman Strengths and Difficulties Questionnaire (Goodman, 1997). Questions included “child is sensitive to others’ feelings” “happy to share things with other children” “happy to help if someone is hurt, depressed, or sick” and so on. The questionnaire is rated on a scale of 1–5. The questionnaire was rated on a scale of 1–5, from 1 “not at all” to 5 “very much,” and the average score was taken as the final score, with higher scores indicating higher levels of prosocial behavior. The Cronbach’s alpha coefficient for this questionnaire was 0.812.

### The authoritative parenting style

The authoritative parenting style was adopted from the Parenting Style Questionnaire developed by Robinson et al. and consists of three dimensions: warmth and involvement dimension, reasoning/induction dimension, and democratic participation dimension, with 23 items (Robinson et al., 1995). Questions include “I praise my child when he or she behaves well” “I give my child reasons for following rules” “I take my child’s preferences into account when making family plans” and so on. The questionnaire was rated on a scale of 1 to 5, from 1, “not very much,” to 5, “very much,” and the average score was taken as the final score, with higher scores indicating higher levels of authoritative parenting. The Cronbach’s alpha coefficient for this questionnaire was 0.913.

## Results

In this study, the mediation model with moderation was tested using SPSS version 25.0 and Hayes’ PROCESS version 4.0. First, descriptive statistics were analyzed using SPSS, and means and standard deviations between the main variables were calculated. In addition, the relationships between parents phubbing, closeness child–parent relationship, preschoolers’ prosocial behavior, and authoritative parenting style were assessed using Pearson’s correlation. Second, the mediating effect of closeness child–parent relationship was tested by Model 4 of PROCESS, and the moderating effect of authoritative parenting style was tested by Model 59 of PROCESS.

### Common method bias

Since the data rely on Parent’ subjective self-reports, there may be some covariations, which means that common method bias needs to be examined. First, We designed the questionnaire using the basic layout method as well as the anonymous response format with reverse scoring questions. In addition, the Harman single factor test was used to determine the common method deviation or systematic measurement error (Harman, 1976). As shown by the findings, six factors had eigenvalues greater than 1, and the first factor of the amount of variation explained was 22.66%, which is below the threshold criterion of 40% (Podsakoff et al., 2003). Thus, the common method bias in this study was not so strong to influence the relationship between variables.

### Descriptive and correlation statistics

The descriptive and correlation analysis of the major variables are provided in Table 2. The results showed that significantly negatively correlations between parents pubbing and children’s prosocial behavior; closeness child–parent relationship and parents pubbing had negative correlations. Additionally, there was a positive correlation between closeness child–parent relationship, authoritative parenting style and children’s prosocial behavior. Parents pubbing was negatively associated with authoritative parenting style. The study also demonstrated that there was a significant correlation between parents pubbing, children’s prosocial behavior and age, and closeness child–parent relationship, children’s prosocial behavior and gender. In addition, the VIF values of each variable is less than 10, which shows that there is no problem of multicollinearity between the variables.

**Table 2:** Means, standard deviations, and correlations of the variables (*N* = 3483).

Variables	M	SD	1	2	3	4	5	6	VIF
Age	3.791	0.669	1						1.008
Gender	3.978	0.564	0.060	1					1.003
Parents phubbing	1.490	0.500	0.063^**^	−0.020	1				1.010
Closeness child–parent relationship	2.280	0.955	0.007	0.051^**^	−0.043^*^	1			1.821
Authoritative parenting style	3.857	0.613	−0.046^**^	0.025	−0.078^**^	0.669^**^	1		1.828
Prosocial behavior	2.524	0.633	0.100^**^	0.110^**^	−0.063^**^	0.659^**^	0.590^**^	1	

*p < 0.05, **p < 0.01.

### Results of the mediating effect of closeness child–parent relationship

This study used Model 4 in the SPSS PROCESS macro by Hayes (2012) to assess the mediating roles of parents phubbing and children’s prosocial behavior. All data were processed and transformed into Z-scores. The results (refer to Tables 3, 4) showed a significant negative correlation between parents phubbing and prosocial behavior in preschool children (*β* = −0.73, *t* = −4.096, *p* < 0.01). And when mediating variables were put in, the relationship between parents phubbing and children’s prosocial behavior remained significant (*β* = −0.043, *t* = −3.195, *p* < 0.01). In addition, parents phubbing was a significant negative predictor of closeness child–parent relationship (*β* = −0.042, *t* = −2.565, *p* < 0.01), while closeness child–parent relationship was a significant predictor of children’s prosocial behavior (*β* = 0.712, *t* = 51.494, *p* < 0.05). Furthermore, the upper and lower limits of the bootstrap 95% CI for the direct effect of parents phubbing on children’s prosocial behavior and the mediating effect of closeness child–parent relationship did not contain 0 (refer to Table 4), indicating that parents phubbing can directly and negatively predict children’s prosocial behavior through closeness child–parent relationship. The direct effect (−0.043) and the mediating effect (−0.030) respectively accounted for 58.9 and 41.1% of the total effect (−0.073).

**Table 3:** The mediation model of child–parent relationship.

Regression equation (*N* = 999)		Fitting index			Coefficient significance	
Outcome variable	Predictor variable	*R*	*R* ^2^	F(df)	*β*	*t*
Prosocial behavior	Parents phubbing	0.164	0.027	31.640 (3)	−0.730	−4.096^**^
Closeness child–parent relationship	Parents phubbing	0.068	0.005	5.263 (3) ^**^	0.042	−2.565^**^
Prosocial behavior	Closeness child–parent relationship Parents phubbing	0.672	0.451	704.969 (4) ^**^	0.712 −0.043	51.494^**^ −3.195^**^

**p < 0.01.

**Table 4:** Analysis of total effect, direct effect, and mediating effect.

	Effect	Boot SE	Boot LLCI	Boot ULCI	Percentage of in effect value
Total effect	−0.073	0.018	−0.038	−0.069	
Direct effect	−0.043	0.013	−0.176	−0.041	58.9%
Mediating effect of closeness child–parent relationship	−0.030	0.013	−0.057	−0.040	41.4%

### Results of the moderation mediating model

**Figure 2:**
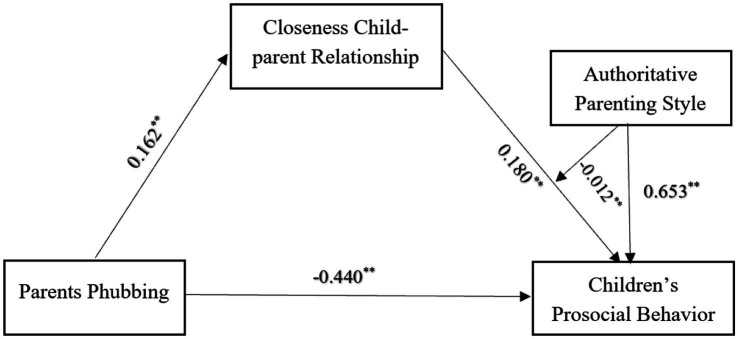
The moderated mediation model. **p < 0.01.

The study examined the moderating effect of authoritative parenting style through Hayes’ PROCESS macro (model 59). It was hypothesized that moderator influenced the three paths of the mediation model, and the actual paths of authoritative parenting style were further determined based on the results of data analysis. The results (refer to Table 5) suggests that authoritative parenting style has significantly moderating effect between closeness child–parent relationship and children’s prosocial behavior (*β* = −0.102, *t* = −2.607, *p* < 0.01). Authoritative parenting style has no significant moderating effect between parents phubbing and closeness child–parent relationship (*β* = −0.011, *t* = −0.674, *p* > 0.01) and between parents phubbing and children’s prosocial behavior (*β* = −0.005, *t* = −0.171, *p* > 0.01). This result showed that authoritative parenting style can only play a moderating role between closeness child–parent relationship and children’s prosocial behavior (refer to Figure 2).

**Table 5:** The moderated mediation model analysis.

Regression equation (*N* = 999)		Fitting index			Coefficient significance	
Outcome variable	Predictor variable	*R*	*R^2^*	*F(df)*	*β*	*T*
Closeness child–parent relationship	Parents phubbing Authoritative parenting style Parents phubbing×Authoritative parenting style	0.406	0.165	226.295 (3)^**^	0.162 0.215 −0.011	16.478 20.747^**^ −0.674
Prosocial behavior	Parents phubbing Closeness child–parent relationship Authoritative parenting style Parents phubbing×Authoritative parenting style Closeness child–parent relationship× Authoritative parenting style	0.598	0.358	382.173 (5) ^**^	−0.440 0.180 0.653 −0.005 −0.102	−2.694^**^ 6.639^**^ 37.067^**^ −0.171 −2.607^**^
		

**p < 0.01.

**Figure 3:**
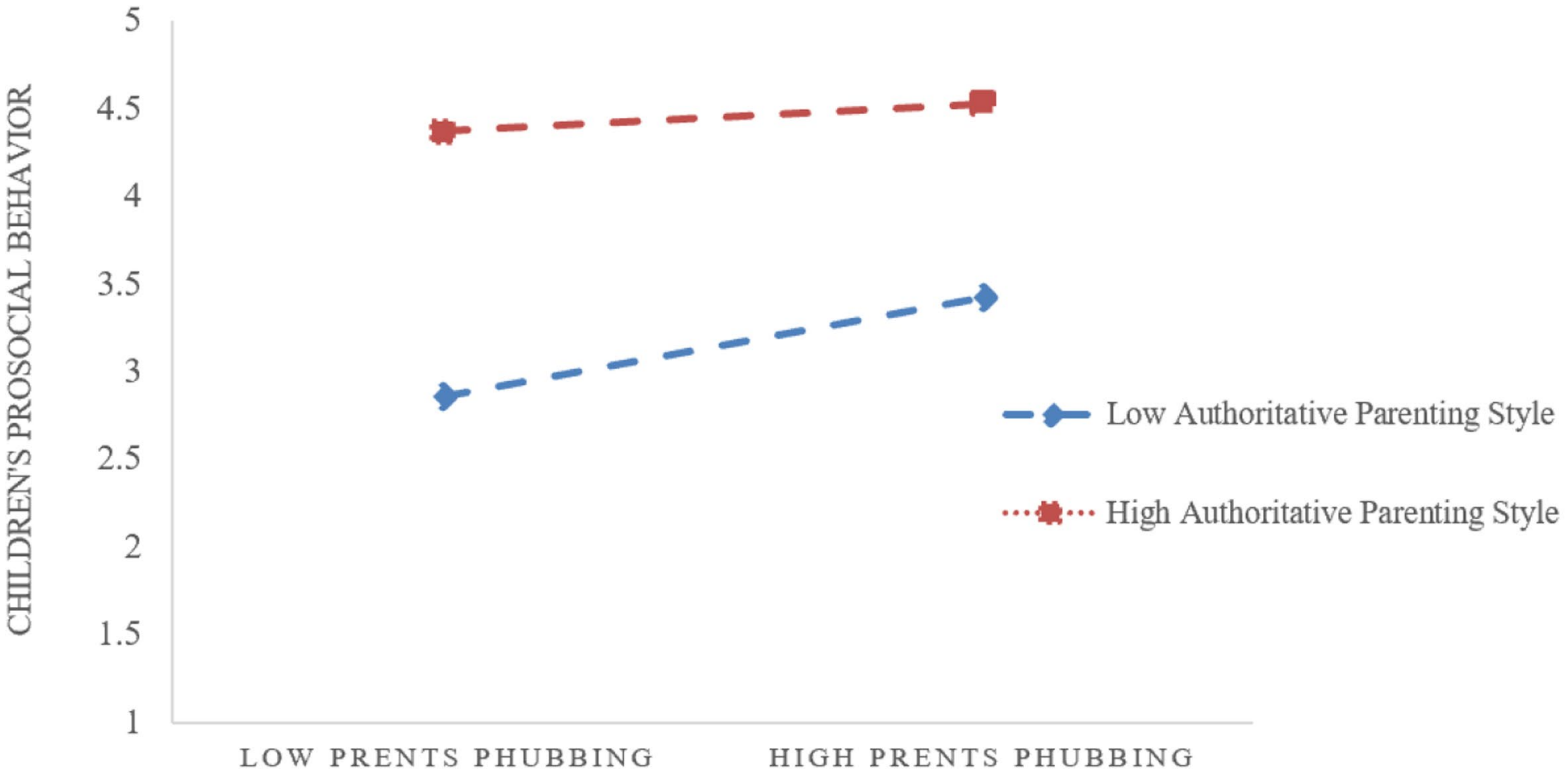
The moderating effect of authoritative parenting styles.

To more clearly reveal the moderating effect of authoritative parenting style, the study further conducted a simple slope test (refer to Figure 3). The results showed that as the level of authoritative parenting style increased, the relationship between closeness child–parent relationship and children’s prosocial behavior became stronger (simple slope = 0.237, *t* = 6.444, *p* < 0.01). In addition, the mediating effect of closeness child–parent relationship tended to decrease at all three levels of authoritative parenting styles (refer to Table 6). As the level of authoritative parenting styles increased, parents phubbing was less likely to influence children’s prosocial behavior by affecting the closeness child–parent relationship.

**Table 6:** Direct effects on different levels of authoritative parenting style.

	Authoritative parenting style	Effect	Boot SE	Boot LLCI	Boot ULCI
Direct effect	−1 (M-1SD) 0 (M) 1 (M + 1SD)	0.040 0.029 0.019	0.008 0.005 0.006	0.024 0.019 0.008	0.057 0.040 0.031
The mediating role of Closeness child–parent relationship	−1 (M-1SD) 0 (M) 1 (M + 1SD)	−0.021 −0.011 −0.010	0.005 0.009 0.004	−0.021 −0.038 −0.017	−0.001 −0.002 −0.001

## Discussion

### The association between parents phubbing and preschool children’s prosocial behavior

The results of the study showed that there was a significant negative correlation between parents phubbing and prosocial behavior of preschool children. In other words, parents phubbing will be detrimental to the development of prosocial behavior in preschoolers. This view supports most of the current research (Vanden Abeele et al., 2020; Pancani et al., 2021; Solecki, 2022). Children may feel neglected and isolated when their parents look down at a screen, and this sense of isolation may lead to a diminished interest in social interactions and reduce children’s motivation to develop prosocial behaviors (Hong et al., 2019). In addition, children often mimic the behavior of their parents or primary caregivers, and when parents overuse mobile phones or electronic devices, children also focus their interest on the screen and lack interaction with and learning from their peers. Therefore, parents phubbing may deprive children of positive social role models, thus affecting their social development. In addition to this, the language and emotional development of preschool children is closely related to parent–child interactions (Menashe and Atzaba-Poria, 2016). When parents look down at a screen, they are often unable to communicate effectively with their children, which can lead to suppression of the child’s language skills as well as weakening the emotional connection between parent and child. Preschool children need emotional support and guidance from their parents to establish positive prosocial behavior, but this support will be insufficient when parents’ attention is turned to screens. In conclusion, parents should strengthen communication and exchange with their children to reduce the occurrence of parents phubbing and promote the development of prosocial behavior.

### Mediating effect of closeness child–parent relationship

Closeness child–parent relationship mediates the relationship between parents phubbing and preschoolers’ prosocial behavior. That is, parents phubbing affects preschoolers’ prosocial behavior by influencing closeness child–parent relationship and, in turn, preschoolers’ prosocial behavior. First of all, parents phubbing has a negative impact on closeness child–parent relationship. This finding supports related studies (Mackay et al., 2022; Frackowiak et al., 2023). On the one hand, when parents spend most of their time on screens, children may feel that they need to compete with their electronic devices for their parents’ attention, and this competition for attention can lead to tension and conflict in the parent–child relationship, hindering the development of a sense of intimacy. On the other hand, children need to feel emotionally supported and cared for by their parents, and parents phubbing may trigger insecurity and anxiety in children, negatively affecting the intimate child–parent relationship. In addition to this, parents devote most of their time to screens rather than engaging in activities with their children. This may lead to children feeling deprived of the opportunity to spend time with their parents, thus affecting the quality of the child–parent relationship (Ganotice et al., 2017).

Secondly, closeness child–parent relationships influence the development of prosocial behavior in preschool children. This finding supports many studies (Feldman, 2007; Pallini et al., 2014; Saral and Acar, 2021). Closeness child–parent relationships provide an important foundation upon which children can build a sense of emotional security. When children feel that they are loved, understood and accepted by their parents, they are more likely to build self-esteem and confidence (Calatrava et al., 2023). In addition, closeness child–parent relationships help to develop children’s emotional intelligence, enabling them to understand and process their own and others’ emotions. This emotional intelligence helps children to get along better with others and to respond positively to the emotional needs of others, thereby displaying more prosocial behavior. Furthermore, when children know they can rely on their parents to meet their emotional needs, they feel more confident to explore the outside world and interact with others. This trust and attachment helps children overcome social challenges and exhibit more prosocial behavior.

### Moderating effect of authoritative parenting style

According to the results of the study, the mediating effect of closeness child–parent relationship tended to decrease at all three levels of authoritative parenting styles. In other words, as the level of authoritative parenting styles increases, the impact of parents phubbing on children’s prosocial behavior tends to weaken. This is in keeping with the findings of related studies (Pinquart, 2017; Eti, 2023). On the one hand, the authoritative parenting style encourages the establishment of a positive and intimate relationship between parents and children. This establishment of intimacy helps preschoolers to feel loved and accepted by their parents, thus increasing their sense of emotional security (Wang et al., 2022). On the other hand, authoritative parents are usually willing to listen to their children’s feelings and needs while providing a safe environment for them to explore their emotional world. This emotional support helps preschoolers to develop a sense of emotional security, which in turn makes them more willing to actively participate in society (Mortazavizadeh et al., 2022). In addition, authoritative parenting styles emphasize clear rules and boundaries. This clarity helps to reduce conflict and confusion and improves the stability of the family atmosphere. It also teaches children the importance of social behavior and develops their social skills.

## Limitations and directions

There are still some limitations to this study. First, the source of data was only parent self-reported data, which may lead to some sample bias in the study. Due to the limitation of children’s age, this study could only collect relevant data by distributing parent questionnaires, but this practice will have some bias, which will reduce the validity of the findings. Therefore, there is a need to minimize bias and increase reliability by adopting a variety of measures, such as third-party observation. Second, the study involves limited core variables. The main purpose of this study is to investigate the impacts of parents phubbing on preschoolers’ prosocial behavior. Meanwhile, closeness child–parent relationship and authoritative parenting style were used as mediating and moderating variables, respectively, wanting to clarify the relationship between the variables through constructive modeling. However, there are many factors related to the influence of prosocial behavior in preschool children, which can only be explored in a limited way at present, and more factors will be included in the future to clarify the relevant influence mechanisms. Third, cross-sectional studies could not establish causality, and longitudinal and experimental studies are needed to confirm these associations. A series of follow-up studies may be needed in the future to continually verify causal associations between variables and clarify internal mechanisms of action. Finally, Zhuhai is a well-developed city in China, so generalization may be another limitation of this study.

## Conclusion

This study explored the association between parents phubbing and prosocial behavior in preschool children. The findings indicated that parents phubbing had a significant negative relationship with prosocial behavior in preschool children. Among them, closeness child–parent relationship played a mediating effect, while authoritative parenting style moderated the relationship between closeness child–parent relationship and preschoolers’ prosocial behavior. From the theoretical level, our study clarifies the mechanism of parents phubbing’s influence on preschool children’s prosocial behavior, which is helpful for us to understand the antecedents of preschool children’s prosocial behavior. Meanwhile, this study enriches theories related to preschool children’s social development. On a practical level, our study is an important guide for parenting. According to the results of this study, parents should reduce the occurrence of phubbing in front of their children and cultivate more closeness child–parent relationship, which will be beneficial to the development of preschool children’s prosocial behavior.

## Data availability statement

The raw data supporting the conclusions of this article will be made available by the authors, without undue reservation.

## Ethics statement

The studies involving humans were approved by Ethics Committee of Minzu University of China. The studies were conducted in accordance with the local legislation and institutional requirements. Written informed consent for participation in this study was provided by the participants’ legal guardians/next of kin.

## Author contributions

DS: Writing – original draft. YX: Writing – review & editing. LC: Writing – review & editing.
